# Liver abscess and bacteremia caused by lactobacillus: role of probiotics? Case report and review of the literature

**DOI:** 10.1186/s12876-016-0552-y

**Published:** 2016-11-18

**Authors:** Muhammed Sherid, Salih Samo, Samian Sulaiman, Husein Husein, Humberto Sifuentes, Subbaramiah Sridhar

**Affiliations:** 1 Section of Gastroenterology and Hepatology, Georgia Regents University, 1120 15th Street-AD 2226, Augusta, GA USA; 2 Department of Medicine, Northwestern Memorial Hospital, Northwestern University Feinberg School of Medicine, 251 East Huron Street, Suite 16-738, Chicago, IL 60611 USA; 3 Department of Internal Medicine, Froedtert Hospital & Medical College of Wisconsin, 9200 West Wisconsin Avenue, Milwaukee, WI 53226 USA; 4 Department of Internal Medicine, Seton Hall University, School of Health and Medicine Sciences, Trinitas Regional Medical Center, 225 Williamson Street, Elizabeth, NJ 07202 USA

**Keywords:** Liver abscess, Lactobacillus, Probiotics, Cholecystectomy

## Abstract

**Background:**

Lactobacilli are non-spore forming, lactic acid producing, gram-positive rods. They are a part of the normal gastrointestinal and genitourinary microbiota and have rarely been reported to be the cause of infections. Lactobacilli species are considered non-pathogenic organisms and have been used as probiotics to prevent antibiotic associated diarrhea. There are sporadic reported cases of infections related to lactobacilli containing probiotics.

**Case presentation:**

In this paper we discuss a case of an 82 year old female with liver abscess and bacteremia from lactobacillus after using probiotics containing lactobacilli in the course of her treatment of *Clostridium difficile* colitis. The Lactobacillus strain identification was not performed and therefore, both commensal microbiota and the probiotic product should be considered as possible sources of the strain.

**Conclusion:**

Lactobacilli can lead to bacteremia and liver abscesses in some susceptible persons and greater awareness of this potential side effect is warranted with the increasing use of probiotics containing lactobacilli.

## Background

Lactobacilli are facultative anaerobic, non-spore forming, lactic acid producing, and Gram positive bacilli. They are found in the normal microbiota of the oral cavity, gastrointestinal tract, and female genitourinary tract. Although lactobacilli are generally considered non-pathogenic microbes and some of their strains are utilized as probiotics to prevent and treat some infections, they have been implicated in some serious clinical infections including bacteremia, infective endocarditis, intra-abdominal abscess including liver abscess, pancreatic necrosis infection, pulmonary infections, pyelonephritis, meningitis, postpartum endometritis, and chorioamnionitis [1–14].

The most common risk factors for lactobacilli infections that have been reported in the literature are diabetes mellitus, pre-existing structural heart disease (in infective endocarditis cases), cancer (especially leukemia), total parenteral nutrition use, broad spectrum antibiotic use, chronic kidney disease, inflammatory bowel disease, pancreatitis, chemotherapy, neutropenia, organ transplantation (especially liver transplantation), HIV infection and steroids use [1–14]. The most common predisposing events are dental manipulation, poor dental hygiene, intravenous drug abuse, abdominal surgery, colonoscopy, probiotics use, and heavy dairy product consumption [1–12].

The antibiotic susceptibility of lactobacilli is variable. The most common regimens that have been used to treat lactobacilli are high dose penicillin and ampicillin with or without aminoglycosides. In a retrospective study of 45 cases of lactobacilli bacteremia, the bacteria were susceptible to ampicillin (100%), penicillin (96%), clindamycin (100%), erythromycin (100%), and gentamycin (67%), with high resistance rate to vancomycin (73% were resistant) [5]. Due to rarity of the infections with this microbe, clinical experience and studies are lacking regarding the best antibiotic regimens.

The incidence of serious infections caused by lactobacilli is generally uncommon and rare with liver abscess with only 7 reported cases in the literature [14–20]. A previous case of liver abscess caused by a lactobacillus strain (indistinguishable from the strain used in food preparation) has been reported. In that case, there was a four month history of excessive dairy products consumption before the development of liver abscess with no use of probiotics. The association between liver abscess and use of probiotics containing lactobacilli has not been reported previously [19].

In this paper, we describe a case of liver abscess due to lactobacillus strain in an elderly diabetic woman with end stage renal disease after using probiotics containing lactobacilli in a course of treatment of *Clostridium difficile* colitis and review the literature. We followed CARE reporting guidelines in publishing our case report.

## Case presentation

An 82 year old female with a history of diabetes mellitus, hypertension and end stage renal disease presented with a 4 day history of generalized weakness, malaise and anorexia. Her symptoms were associated with low grade fever, nausea and vomiting. She denied abdominal pain, chest pain, cough, or shortness of breath. She had recently been diagnosed with *Clostridium difficile* colitis and had been treated with a 2 week course of metronidazole and probiotics containing lactobacilli. Her past surgical history was significant for a cholecystectomy which was complicated by bleeding in the gallbladder fossa and required laparoscopic surgery 4 months prior to this presentation.

On physical examination, she was febrile with a temperature of 100.6 °F (38.1 °C). Cardiopulmonary examination revealed decreased breath sounds in the base of right lung. Abdominal examination demonstrated mildly tender hepatomegaly 3 cm below the costal margin, active bowel sounds, no distension and no guarding or rebound tenderness. Extremities and skin examinations were unremarkable.

**Fig. 1:**
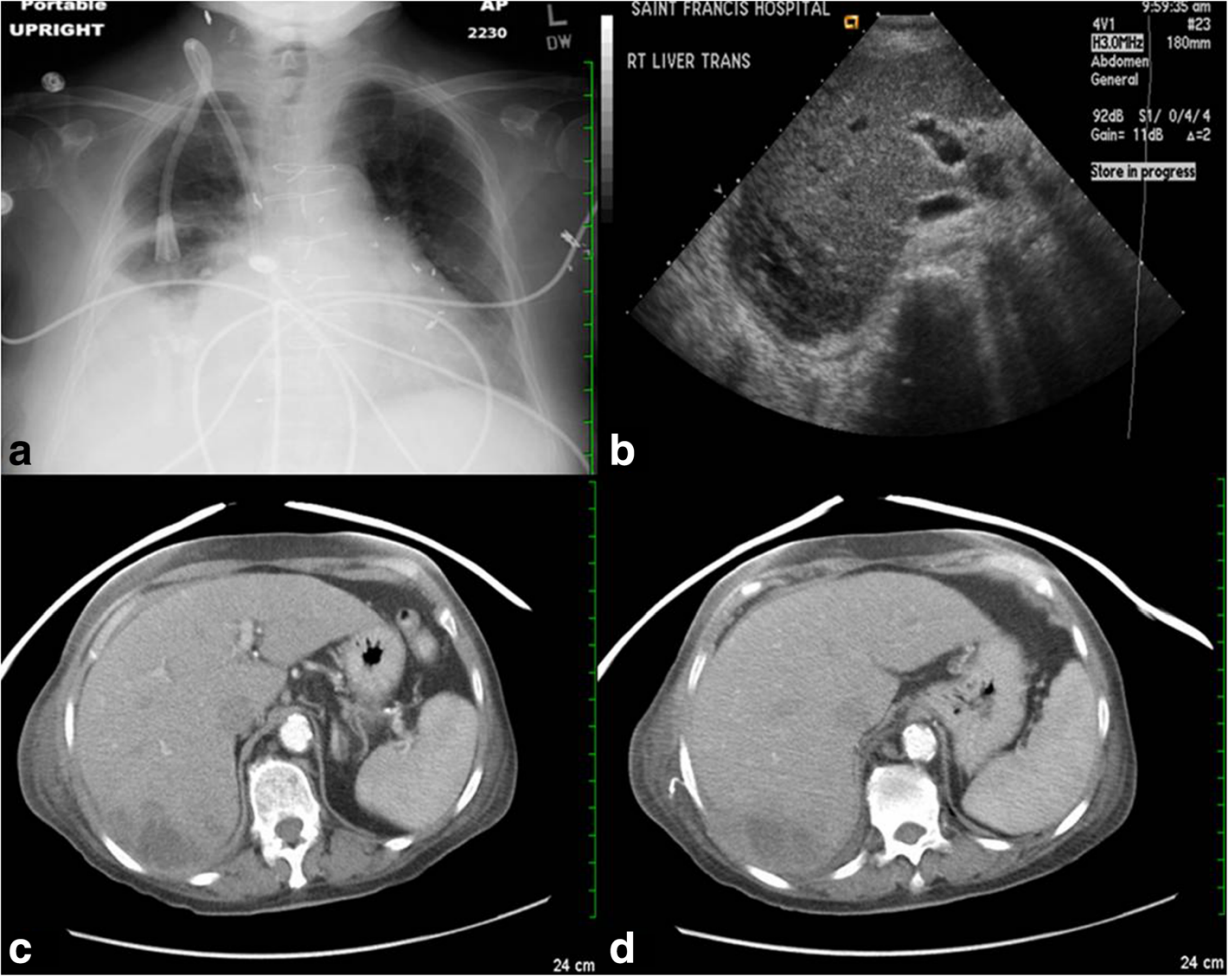
**a** CXR shows elevated right hemi-diaphragm with right lower lobe infiltration and pleural effusion. **b** U/S of RUQ shows hypodensity multiloculated mass in posterior aspect of right hepatic lobe. **c**, **d** CT scan of abdomen shows 6.6 × 4.7 cm hypodensity multiloculated mass in posterior aspect of right hepatic lobe

**Fig. 2:**
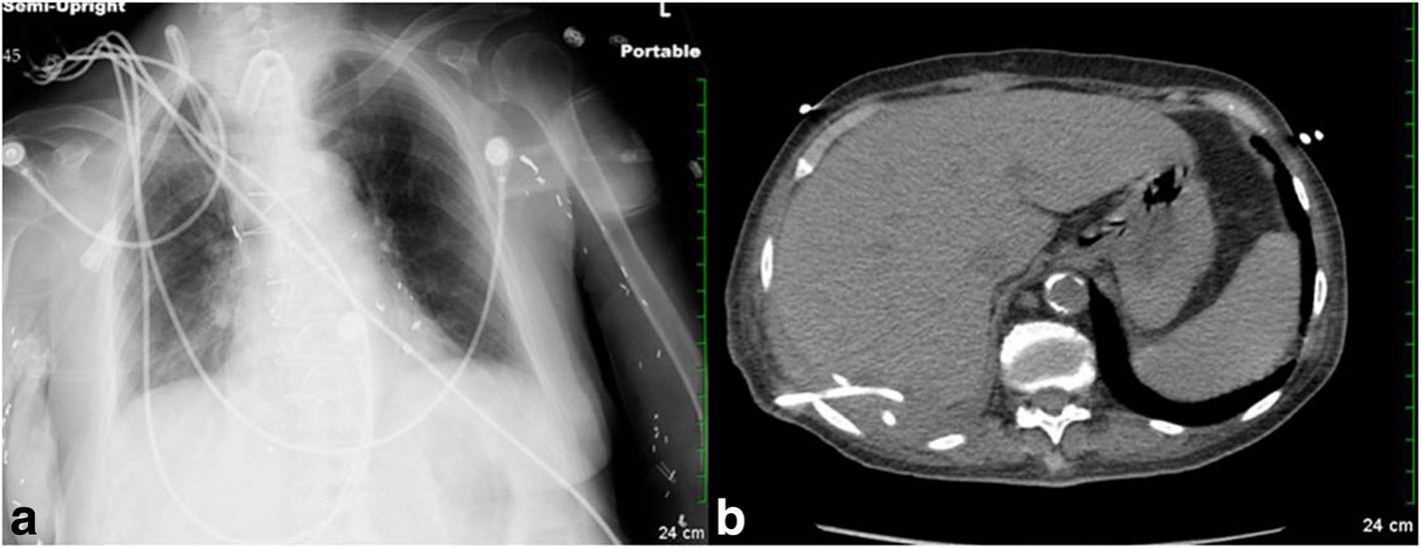
**a** CXR after treatment shows significant improvement comparing to CXR in Fig. 1a. **b** CT scan of abdomen shows significant improvement with pigtail catheter in place

Laboratory studies revealed a leukocytosis of 18.6 k/mm^3^ (normal range: 4–10 k/mm^3^ ), microcytic hypochromic anemia with a hemoglobin of 9.8 g/dl (12–16 g/dl), elevated alkaline phosphatase of 435 IU/L (44–147 IU/L), mild elevation of AST and ALT (97 and 85 IU/L respectively; normal ranges: 5–40 IU/L) and normal bilirubin. Plain chest X-ray (CXR) showed an elevated right hemidiaphragm with a right lower lobe infiltration and pleural effusion (Fig. 1a). A CT scan of the chest and abdomen showed a 6.6 × 4.7 cm hypodense, multiloculated mass in posterior aspect of right hepatic lobe (Fig. 1c, d). Empiric antibiotics with imipenem and vancomycin were begun. The following day with the use of ultrasound guidance, 20 mL of purulent fluid specimen was aspirated from the liver lesion and a percutaneous pigtail catheter was placed for drainage (Fig. 1b). Gram stain from the aspirated materials showed rare Gram positive cocci, Gram negative rods but predominantly Gram positive rods. The blood cultures grew Gram positive rods. Bacteriologic studies confirmed that they were lactobacilli species from both the liver abscess and blood, however the exact strain identification was not performed. Her hospitalization was complicated with cardiac arrest which she survived. She was hospitalized for 3 weeks with near complete resolution of the hepatic abscess which was demonstrated on repeat imaging (Fig. 2a, b).

## Conclusion

Pyogenic liver abscesses are relatively uncommon, however they are considered the most common type of intra-abdominal abscesses with an annual incidence of 2.3 cases per 100,000 [21, 22]. The risk factors for developing pyogenic liver abscess are advanced age, diabetes mellitus, liver transplantation, underlying hepatobiliary disease and history of malignancy [21–25]. The mortality rate has decreased significantly over last six decades from 65% before the 1970s to 10–12.3% in recent studies [21–25]. Independent risk factors associated with mortality are significant delay in diagnosis, severity of sepsis, presence of gas forming bacteria, presence of anaerobic infection, hepatopancreaticobiliary malignancy and requirement for open surgical drainage [24, 25].

Liver abscesses usually result from seeding of bacteria from the portal vein or biliary tract; however, hematogenous spread from systemic circulation or direct seeding from penetrating wounds may be the main mechanism in other cases [22, 25]. A considerable proportion of liver abscesses result from polymicrobial aerobic and anaerobic bacteria with some differences based on the underlying disease. The geographic area may also play a role, for example klebsiella pneumoniae is the most common isolated bacteria in Hong Kong [22, 23, 25].

Pyogenic liver abscess due to lactobacillus has rarely been reported in the literature. Our case developed in an elderly diabetic woman after four months of a cholecystectomy which was complicated by bleeding from the surgical site which required surgical drainage. Whether she had bacterial seeding at the time of the surgery or had bacterial translocation from the colon from probiotic bacteria or intestinal microbiota is not clear.

We used the MeSH database in Pubmed with the terms “lactobacillus” and “liver abscess”. We found 7 additional cases of liver abscesses due to lactobacilli strains (Table 1) [14–20]. The median age was 73.5 years (range, 27–82 years), with 62.5% older than 73 years of age. There was no gender tendency. The most common underlying diseases were diabetes mellitus (62.5%) and hepatopancreaticobiliary diseases (50%). Predisposing factors were steroids use, heavy dairy product consumption and intratumoral ethanol injection. In our case, the patient’s recent cholecystectomy and probiotic use were considered predisposing factors. The most frequent presenting symptoms were fever (87.5%), abdominal pain (37.5%), and vomiting (25%). Leukocytosis and elevated liver enzymes were the most common laboratories findings. Lactobacillus rhamnosus was the most frequently isolated strain.

**Table 1:** Summary of case reports of liver abscesses due to lactobacilli strains

Reference	Age (years)/Sex	Comorbidities	Predisposing events	Symptoms	Labs	Organism (site)	Treatment	Hospital stay length	Outcomes
Chan (2010) [16]	74/M	DM, HTN, remote history of tonsillar carcinoma	Mirizzi syndrome (common hepatic duct obstruction secondary to external compression by gallstone)	Fever, abdominal pain for 1 day.	Leukocytosis, normal LFTs.	L. rhamnosus (blood, pus, gallbladder)	Percutaneous drainage, cholecystectomy, antibiotics (levofloxacin then both clarithromycin and metronidazole were added)	59 days	Required ventilator for some time and discharge to rehabilitation.
Burns (2007) [17]	51/F	None	None	Abdominal pain, vomiting for 2 weeks.	Leukocytosis, elevated LFTs.	L.paracasei (pus)	Percutaneous drainage, antibiotics (meropenem with penicillin and gentamicin, then changed to clindamycin)	53 days	Developed purpura fulminans. Required ventilator for some time and discharged.
Cukovic-Cavka (2006) [18]	27/M	Crohn’s disease	Steroid use	Fever, diarrhea and fatigue.	Leukocytosis.	L.acidophilus (blood, pus)	Percutaneous drainage, antibiotics (ciprofloxacin with metronidazole, then Augmentin with metronidazole)	63 days	Discharged.
Notario (2003)* [15]	73/F	DM	N/A	Fever	N/A	L.rhamnosus (blood, pus)	Surgical drainage, antibiotics (ampicillin with gentamicin)	N/A	Discharged.
Rautio (1999) [19]	74/F	DM, HTN	Heavy dairy consumption.	Fever, abdominal pain for 2 weeks.	Leukocytosis.	L.rhamnosus (pus)	Percutaneous drainage, antibiotics (penicillin, then piperacillin/tazobactam, then ciprofloxacin and clindamycin)	42 days	Complicated with pleural empyema which required surgical thoracotomy and decortication. Discharged.
Larvol (1996)* [14]	39/M	DM, chronic pancreatitis, choledochoduodenostomy	N/A	Fever	N/A	L.acidophilus (blood, pus)	Antibiotics (amoxicillin, gentamicin, augmentin)	N/A	Discharged.
Isobe (1990) [20]	75/M	(HCC, Parkinson’s disease	Intratumoral ethanol injection therapy for HCC	Fever	Intrahepatic gas by U/S and CT scan	L.plantarum (blood)	Antibiotics (piperacillin)	52 days (after developing fever)	Discharged.
Sherid (2016) (the current case)	82/F	DM,HTN, ESRD, cholecystectomy	Cholecystectomy, probiotic use	Fever, vomiting	Leukocytosis, elevate LFTs, right pleural effusion	N/A	Percutaneous drainage, antibiotics (imipenem, vancomycin)	19 days	Her hospital course was complicated by cardiac arrest which she survived but required long term ventilator. Discharged to nursing home on ventilator

*M* male, *F* female, *DM* diabetes mellitus, *ESRD* end stage renal disease, *HCC* hepatocellular carcinoma, *HTN* hypertension, *LFTs* liver function tests, *N/A* not available. *In foreign language; some information from English abstracts

Although no mortality occurred in these 8 cases, prolonged hospitalization was a striking feature with a mean hospital stay of 48 days (range, 19–63 days) compared to 16 days in other cases of pyogenic liver abscesses. In a retrospective study of 200 cases of endocarditis caused by lactobacilli the mortality rate was 23% which was higher in cases of polymicrobial bacteremia and use of inadequate antibiotics [1]. It has also been shown that lactobacillus infection is a predictor of severe underlying comorbidities and poor long-term prognosis. In a study by Husni et al. 69% of patients with lactobacilli infections died within 1 year and only one death was attributed directly to lactobacillus bacteremia which was polymicrobial [5].

Some strains of lactobacilli are used as probiotics to, presumably, restore non-pathogenic intestinal microbiota and decrease digestive colonization with pathogenic bacteria. The increasing reports of probiotic related infections have raised concern over the safety of these organisms. The epidemiologic study by Salminen et al. published in 2002 did not show an increase in lactobacillus bacteremia after wide use of lactobacillus rhamnosus GG as a probiotic which was introduced to Finland in 1990 [26]. In this study, the average annual incidence of lactobacilli bacteremia was 0.29 cases per 100,000 during the 11 year study period [26]. An increase in nosocomial infections in intensive care pediatric patients and an increase in mortalities in severe acute pancreatitis have been documented in probiotic groups in randomized placebo-controlled studies [27, 28]. In a systemic review of the safety of probiotics in 2010 including 53 trials in which 4131 patients received probiotics, all but three trials showed no increase in complications related to using probiotics [29].

The potential pathogenicity of lactobacilli might come from several mechanisms including the ability of some strains to bind to intestinal mucosa which may play a role in translocation of lactobacilli into the bloodstream; their ability to adhere to extracellular matrix proteins such as collagen; their ability to aggregate platelets; their production of certain enzymes such as glycosidases and proteases which may help to breakdown the glycoproteins of affected tissues [30–32]. In vitro and in vivo experimental studies have shown that some strains are more resistant than others to intracellular killing by macrophages and by the bactericidal effects of nitric oxide [33].

Several cases of lactobacilli related infections have been reported in pediatric and adults using probiotics containing lactobacilli [34–42]. The underlying diseases were preexisting heart disease, HIV infection, organ transplantation, diabetes mellitus, malignancy, and short gut syndrome [34–42]. There were no cases of liver abscesses which makes our case the first case of lactobacillus liver abscess linked to probiotics use.

In our case, the inflamed colonic mucosa due to *Clostridium difficile* colitis may have allowed the translocation of intestinal microbiota lactobacilli or probiotic lactobacilli to the bloodstream and colonized the previously damaged tissue in liver and gallbladder site. The exact Lactobacillus identification and antibiotic sensitivity were not performed, as well as the culture from the probiotic product. Whether the probiotics product contained the identical strain of Lactobacillus as the patient’s abscess is not known. However, the temporal relationship between use of the probiotics containing Lactobacillus and development of liver abscess with Lactobacillus, make the probiotic product a possible source.

In conclusion, probiotics may lead to bacteremia and liver abscesses in some susceptible persons and greater awareness of this potential side effect is warranted with the increasing use of probiotics containing lactobacilli.

## Abbreviation

HIV: Human immunodeficiency virus
